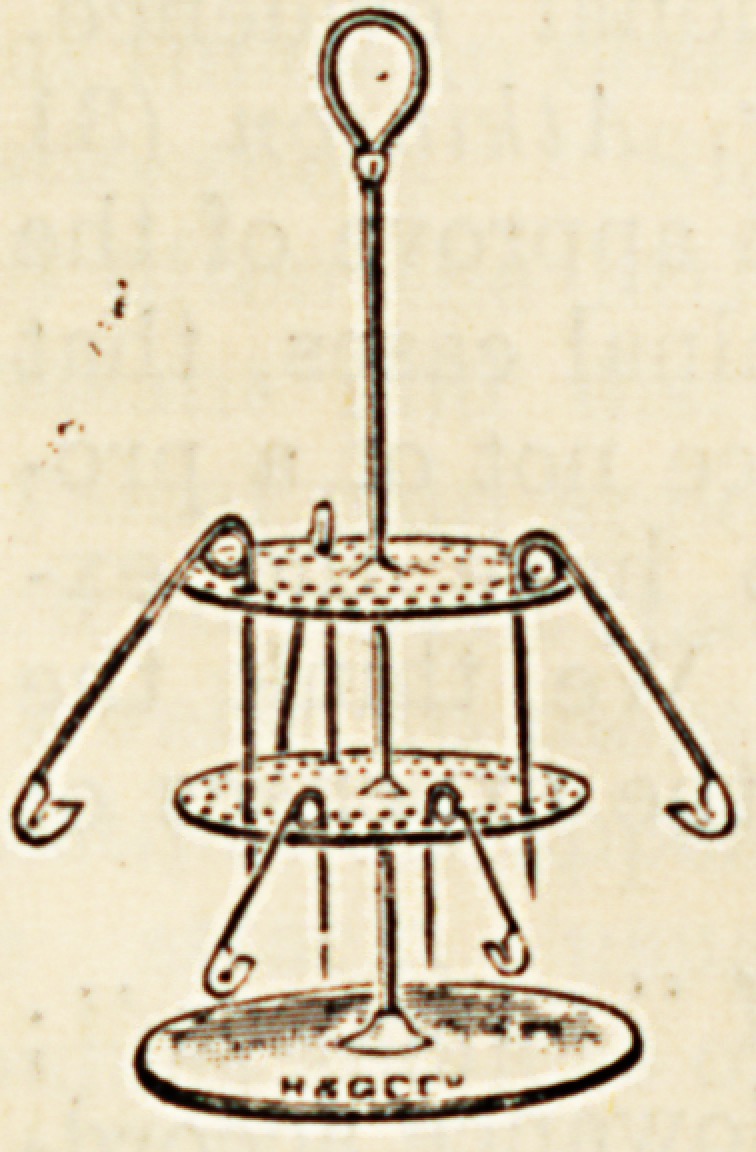# New Appliances and Things Medical

**Published:** 1908-07-25

**Authors:** 


### NEW APPLIANCES AND THINGS MEDICAL.

[We shall be glad to receive at our Office, 28 & 29 Southampton Street, Strand, London, W.C., from the manufacturers, specimens of all new preparations and appliances which may be brought out from time to time.]

### A SAFETY-PIN STERILISER.

"The Gordon Safety-Pin Cruet," for the purpose of sterilising safety-pins, is the invention of a nurse. As its name implies, it resembles a small metal cruet, and it consists of a central rod to which is attached a circular stand and two circular discs, perforated for the reception of the pins. The illustration shows quite clearly the principle of this simple and ingenious appliance. The height is less than six inches, the diameter of the base two inches, and the whole "cruet" is nickel-plated. By standing the cruet in boiling water a large number of safety-pins of any size can be made aseptic with a minimum of trouble. When sterilised in this way the pins are readily accessible to the surgeon, without the intervention of an instrument-holder or another pair of hands. Safety-pins are inconvenient things to render aseptic in ordinary sterilisers, and they have a habit of disappearing from sight at the time when they are wanted. This appliance should therefore be found of service in operating theatres. The price is 3s. 6d. (postage 2d.), and the sole makers are the Hospitals and General Contracts Company, Limited, 33 and 35 Mortimer Street, London, W.

### "FLASH" ANTISEPTIC HAND-CLEANER.

This is a useful substance, hailing from America, which has become popular with engineers and motorists, and is now offered to the notice of the medical profession. It is compounded of glycerine and neutral oil and a small proportion of soap-stock, together with finely-ground pumice. It also contains an essential oil, to which it owes some of the antiseptic properties claimed for it. We have found it a rapid and effectual remover of grease and dirt and stains of various kinds. Since it contains no acid and little if any free alkali, its effect upon the skin is no more injurious than that of finely powdered pumice-stone, and it is therefore preferable to many hand-cleansers. An important point for motorists is that it can be used with cold water. For cleansing the hands before operations, etc., it should prove useful, since it speedily removes dirt of the most obstinate kind from cracks and other hiding-places. "Flash" Hand-cleaner is supplied in this country by Brown Brothers, Limited, 24-30 Great Eastern Street, London, E.C. It is retailed in round tins at 7½d. each.

## Figures and Tables

**Figure f1:**